# Genomic Characterization of Metformin Hepatic Response

**DOI:** 10.1371/journal.pgen.1006449

**Published:** 2016-11-30

**Authors:** Marcelo R. Luizon, Walter L. Eckalbar, Yao Wang, Stacy L. Jones, Robin P. Smith, Megan Laurance, Lawrence Lin, Paul J. Gallins, Amy S. Etheridge, Fred Wright, Yihui Zhou, Cliona Molony, Federico Innocenti, Sook Wah Yee, Kathleen M. Giacomini, Nadav Ahituv

**Affiliations:** 1 Department of Bioengineering and Therapeutic Sciences, University of California San Francisco, San Francisco, California, United States of America; 2 Institute for Human Genetics, University of California San Francisco, San Francisco, California, United States of America; 3 Department of General Biology, Institute of Biological Sciences, Federal University of Minas Gerais, Belo Horizonte, Minas Gerais, Brazil; 4 School of Pharmaceutical Sciences, Tsinghua University, Beijing, China; 5 Library and Center for Knowledge Management, University of California San Francisco, San Francisco, California, United States of America; 6 Bioinformatics Research Center, North Carolina State University, Raleigh, North Carolina, United States of America; 7 Eshelman School of Pharmacy, University of North Carolina at Chapel Hill, Chapel Hill, North Carolina, United States of America; 8 Merck Research Labs, Merck & Co. Inc., Kenilworth, New Jersey, United States of America; The University of Alabama in Huntsville, UNITED STATES

## Abstract

Metformin is used as a first-line therapy for type 2 diabetes (T2D) and prescribed for numerous other diseases. However, its mechanism of action in the liver has yet to be characterized in a systematic manner. To comprehensively identify genes and regulatory elements associated with metformin treatment, we carried out RNA-seq and ChIP-seq (H3K27ac, H3K27me3) on primary human hepatocytes from the same donor treated with vehicle control, metformin or metformin and compound C, an AMP-activated protein kinase (AMPK) inhibitor (allowing to identify AMPK-independent pathways). We identified thousands of metformin responsive AMPK-dependent and AMPK-independent differentially expressed genes and regulatory elements. We functionally validated several elements for metformin-induced promoter and enhancer activity. These include an enhancer in an ataxia telangiectasia mutated (*ATM*) intron that has SNPs in linkage disequilibrium with a metformin treatment response GWAS lead SNP (rs11212617) that showed increased enhancer activity for the associated haplotype. Expression quantitative trait locus (eQTL) liver analysis and CRISPR activation suggest that this enhancer could be regulating *ATM*, which has a known role in AMPK activation, and potentially also *EXPH5* and *DDX10*, its neighboring genes. Using ChIP-seq and siRNA knockdown, we further show that activating transcription factor 3 (*ATF3*), our top metformin upregulated AMPK-dependent gene, could have an important role in gluconeogenesis repression. Our findings provide a genome-wide representation of metformin hepatic response, highlight important sequences that could be associated with interindividual variability in glycemic response to metformin and identify novel T2D treatment candidates.

## Author Summary

Metformin is among the most widely prescribed drugs. It is used as a first line therapy for type 2 diabetes (T2D), and for additional diseases including cancer. The variability in response to metformin is substantial and can be caused by genetic factors. However, the molecular mechanisms of metformin action are not fully known. Here, we used various genomic assays to analyze human liver cells treated with or without metformin and identified in a genome-wide manner thousands of differentially expressed genes and gene regulatory elements affected by metformin. Follow up functional assays identified several novel genes and regulatory elements to be associated with metformin response. These include *ATF3*, a gene that showed gluconeogenesis repression upon metformin response and a potential regulatory element of the *ATM* gene that is associated with metformin treatment differences through genome-wide association studies. Combined, this work identifies several novel genes and gene regulatory elements that can be activated due to metformin treatment and thus provides candidate sequences in the human genome where nucleotide variation can lead to differences in metformin response. It also enables the identification and prioritization of novel candidates for T2D treatment.

## Introduction

Metformin is the first-line oral therapy for Type 2 Diabetes (T2D) [1], and is also approved for use or used off-label in a variety of other diseases, such as polycystic ovary syndrome [2], gestational diabetes [3], pediatric obesity [4] and cancer [5,6]. Side effects of metformin are mainly gastrointestinal in 20% to 30% of patients, and in very rare cases include lactic acidosis [7]. However, the variability in response is substantial, with ≥30% of patients receiving metformin monotherapy classified as non-responders [8]. The genomic characterization of metformin hepatic response would thus provide novel insights into the mechanisms of metformin action.

The molecular mechanisms of metformin action are not fully known [6,9]. Metformin’s major tissue of action is the liver where it inhibits gluconeogenesis by activating the AMP-activated protein kinase (AMPK) pathway [10,11]. Metformin-induced inhibition of the mitochondrial respiratory chain complex I leads to a reduction in ATP synthesis and to an increase in the cellular AMP:ATP ratio, which is thought to activate AMPK [12]. Activation of AMPK is carried out by upstream kinases such as serine/threonine kinase 11 (STK11/LKB1), and ataxia telangiectasia mutated (ATM) that lead to AMPK phosphorylation in the presence of metformin [13]. AMPK is also known to upregulate the nuclear receptor small heterodimer partner (SHP) upon metformin treatment [14], which inhibits cAMP-response element-binding protein (CREB)-dependent hepatic gluconeogenic gene expression [12,15]. Moreover, the phosphorylation of CREB binding protein (CBP) triggers the dissociation of transcription complexes that inhibit gluconeogenic genes [16]. Metformin was also suggested to inhibit hepatic gluconeogenesis independent of the AMPK pathway, via a decrease in hepatic energy state through a process independent of the transcriptional repression of gluconeogenic genes [17]. Moreover, it was proposed that metformin antagonizes the action of glucagon, thus reducing fasting glucose levels [18].

Genetic variation can play an important role in metformin response, with a heritability of 34% based on genome-wide studies [19]. Metformin is not metabolized and transporters are the major determinants of metformin pharmacokinetics. Missense and promoter variants in transporter genes have been associated with metformin pharmacokinetics [20,21]. Notably, genetic variants in OCT1, the major determinant of metformin uptake in hepatocytes, have been associated with metformin action [22,23]. Transcription factors that modulate the expression of metformin transporters were also associated with changes in metformin treatment outcome [24]. A genome-wide association study (GWAS) found a noncoding single nucleotide polymorphism (SNP) rs11212617 nearby the ataxia telangiectasia mutated (*ATM*) gene to be associated with metformin treatment success [25]. These results, though replicated in some smaller cohorts [26], failed replication in a recent three-stage GWAS which identified an intronic SNP in the glucose transporter, *SLCA2*, associated with better metformin response in multiple ethnically diverse cohorts [27]. Combined, these studies have only been able to explain a small portion of the genetic variability associated with metformin response, suggesting a role for additional genetic determinants.

Here, we aimed to characterize metformin response pathways and regulatory elements in a systematic and genome-wide manner. We performed RNA-seq and ChIP-seq for H3K27ac a known active mark [28], and H3K27me3 a repressive mark [28], on primary human hepatocytes from the same donor treated with the following three conditions: 1) vehicle control, 2) metformin, and 3) metformin and compound C (an AMPK inhibitor [11]). We identified AMPK-dependent and AMPK-independent gene clusters using RNA-seq. We found thousands of peaks that were unique to cells treated with metformin using ChIP-seq. Reporter assays in liver cells identified several promoters and enhancers that were modulated by metformin. Moreover, enhancer assays of a metformin increased H3K27ac ChIP-seq peak that has SNPs in linkage disequilibrium (LD) with the metformin treatment response GWAS lead SNP rs11212617 [25], showed increased enhancer activity for the treatment response associate haplotype. Expression quantitative trait locus (eQTL) liver analysis suggests that two SNPs within this enhancer are associated with increased *ATM* expression. Using CRISPR activation (CRISPRa), we found that in addition to *ATM*, *EXPH5* and *DDX10* could also be its target genes. Further analysis of our top upregulated AMPK-dependent gene, activating transcription factor 3 (*ATF3*), using ChIP-seq and siRNA knockdown showed that it may have an important role in suppression of gluconeogenesis. Our systematic studies highlight important metformin response associated genes and regulatory elements in the liver, providing novel sequence targets that could be associated with the vast variability in response to metformin, and identify novel T2D treatment candidates.

## Results

We acquired induction-qualified cryopreserved human hepatocytes originating from a single male donor (see Methods). To identify their optimal metformin response, we treated these cells with 0.5, 2.5 and 10 mM metformin for 4 and 8 hours and analyzed AMPK α2 Thr172 phosphorylation using Western blots (S1 Fig). We observed the highest phosphorylation levels when the cells were treated with 2.5 mM metformin for 8 hours. These conditions also showed high AMPK activity in hepatocytes in another report [29]. These treatment conditions were thus used for all subsequent assays with these cell lines.

### RNA-seq identifies metformin responsive genes

**Fig 1:**
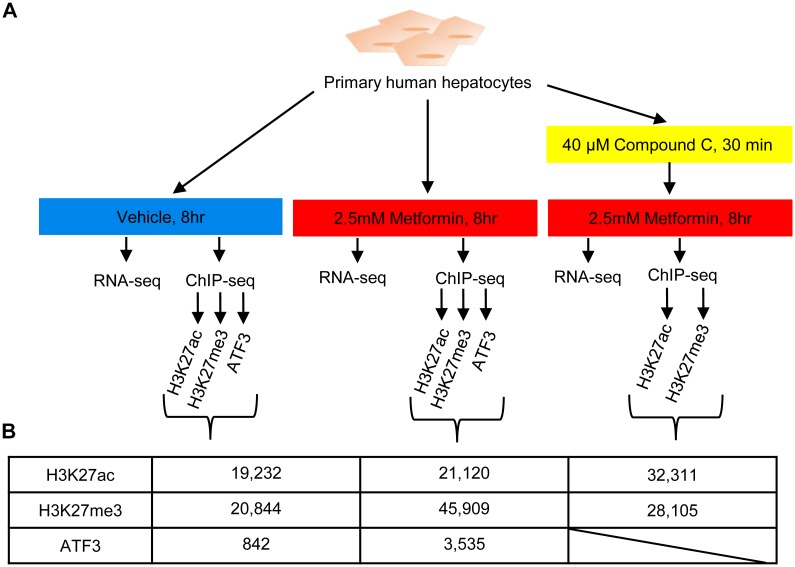
Schematic outline of the study. **(A)** Primary human hepatocytes were treated with either vehicle control, 2.5mM metformin or 40uM compound C + 2.5mM metformin for 8 hours and then subjected to RNA-seq and ChIP-seq. **(B)** Table showing the number of peaks obtained for each antibody and condition.

**Fig 2:**
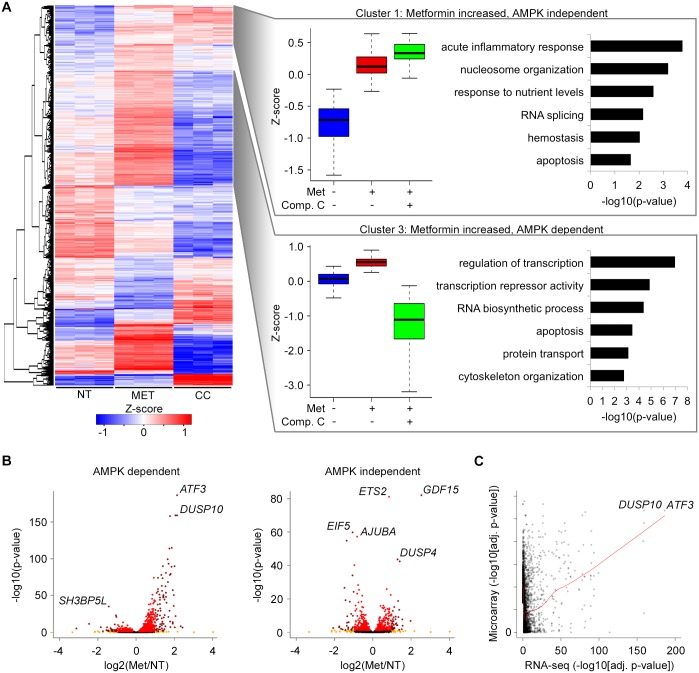
Gene expression profiling of human hepatocytes following treatment with metformin, metformin plus compound C or no treatment control. **(A)** Gene-wise hierarchical clustering heatmap of 1,906 differentially expressed genes in response to metformin treatment (adjusted P ≤ 0.05) showing segregation into 10 groups. The z-score scale represents mean-subtracted regularized log-transformed read counts. Cluster 1 (n = 194) includes genes with increased expression in response to metformin that remain elevated when also treated with compound C (metformin increased, AMPK independent). Cluster 3 (n = 575) includes genes with increased expression in response to metformin that decrease in expression when also treated with compound C (metformin increased, AMPK dependent). Each box in the box plots is the interquartile range (IQR), the line is the median and the whiskers show the furthest data points from the median within 1.5 times the interquartile range. Enriched GO terms are shown to the right. **(B)** Volcano plot displaying fold change versus adjusted p-values of AMPK dependent (left) and AMPK independent (right) genes. **(C)** Comparison of adjusted p-values of genes differentially expressed in response to metformin between this study (RNA-seq, X-axis) and a previously reported microarray experiment (microarray, Y-axis).

To comprehensively identify differentially expressed (DE) genes associated with metformin treatment, we carried out RNA-seq on primary human hepatocytes treated with: 1) vehicle control, 2) 2.5 mM metformin for 8 hours, and 3) 40 μM compound C, an inhibitor of AMPK [11], along with 2.5 mM metformin for 8 hours (Fig 1). Our RNA-seq analyses identified 1,906 DE genes between the vehicle control and both metformin treatment conditions using a p-value cutoff after correction for multiple testing of less than or equal to 0.05 (S1 Table). Amongst them, 1,255 were upregulated and 651 were downregulated (Fig 2A). Notably, we found novel transcription factors related to metformin response, such as the upregulated *ATF3* and *KLF6* and the downregulated *AJUBA* (Fig 2B). Ingenuity pathway analysis (IPA) found networks for upstream regulators enriched for DE genes, further implicating additional molecular pathways to metformin response (S2 Table). We also compared our RNA-seq data with previously reported microarray data from human hepatocytes treated with 1mM metformin for 8 hours [30]. Despite the use of different techniques, conditions, statistical analyses and other variables that could confound these comparisons, we found that 25% of our DE genes overlap with microarray defined DE genes (S2A Fig). Moreover, we observed that several of the highly DE genes are similar in both datasets (Fig 2C) with fold changes showing a Spearman correlation of R^2^ = 0.52 (S2B Fig).

**Fig 3:**
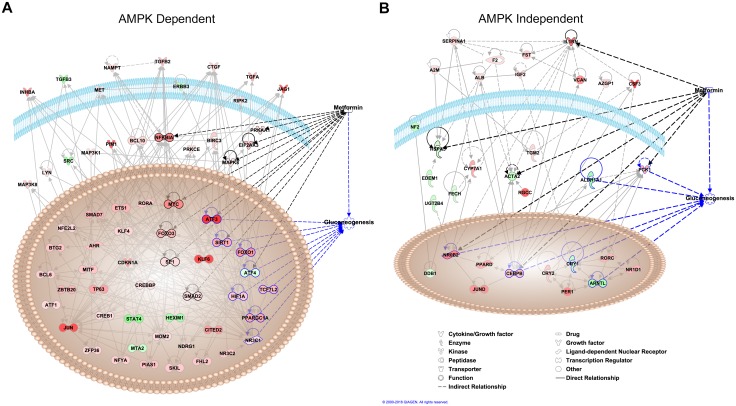
Upstream regulator analysis of RNA-seq data. Transcription factors, kinases, growth factors and other molecules for the **(A)** AMPK-dependent and for the **(B)** AMPK-independent clusters of genes. Upregulated and downregulated genes as determined by RNA-seq are colored in red and green, respectively.

We next generated AMPK-dependent and AMPK-independent clusters by comparing these three conditions (Fig 2A). Of note, as compound C is also thought to have off-target effects [31], we only considered genes whose expression changed due to metformin response for this assay. Clusters two (n = 134), three (n = 575), seven (n = 83) and eight (n = 168) contain genes whose expression increases with metformin treatment, but is greatly reduced with the combined treatment of compound C and metformin. These genes were termed metformin increased, AMPK-dependent. Gene ontology (GO) analysis found enrichment for transcription regulation and several additional terms for these clusters (Fig 2A; S3 Table). Cluster five (n = 256) contains genes whose expression decreases with metformin treatment, but returns to untreated level with the combined treatment of compound C and metformin. These genes were termed metformin decreased, AMPK-dependent and were enriched for ribonucleotide binding (S3 Table). In addition, IPA upstream regulator analysis of AMPK-dependent genes found many transcription factors not previously related to metformin and related to gluconeogenesis, and enrichment for the AMPK signaling canonical pathway (Fig 3A; S3 Fig and S2 Table).

We also identified several AMPK-independent clusters. Of note, Clusters one (n = 194), six (n = 74) and ten (n = 57) contain genes whose expression increases with metformin treatment that remains elevated with the combined treatment of compound C and metformin (Fig 2A; S3 Table). We termed these genes as metformin increased, AMPK-independent. These genes were enriched for acute inflammatory response, chromatin organization and response to nutrient levels and other GO terms (Fig 2A; S3 Table). Clusters four (n = 365) and nine (n = 20) contain genes whose expression decreases with metformin treatment that remains low with the combined treatment of compound C and metformin. These genes were termed metformin decreased, AMPK-independent and were enriched for metabolic processes, RNA processing and other GO terms (S3 Table). IPA upstream regulator analysis of AMPK-independent genes found transcription factors and ligand-dependent nuclear receptors not previously related to metformin, and enrichment for the acute phase response signaling canonical pathway (Fig 3B; S3 Fig and S2 Table).

### ChIP-seq identifies regulatory elements associated with metformin response

**Fig 4:**
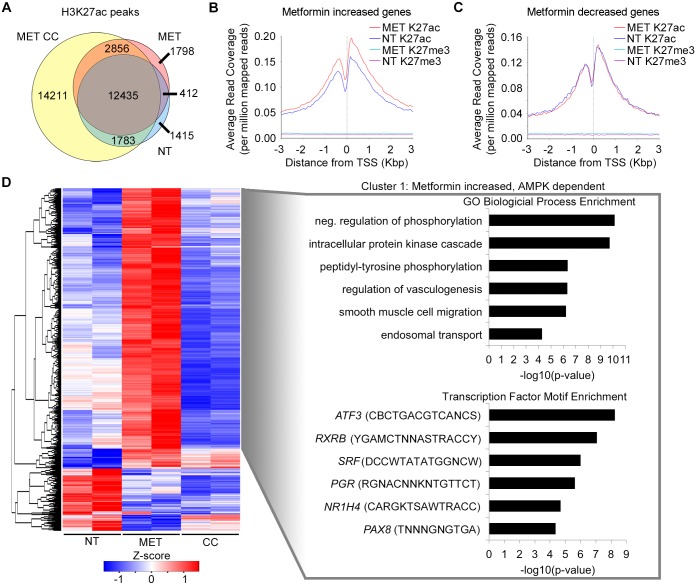
Genome-wide characterization of H3K27ac and H3K27me3 changes in human hepatocytes in response to treatment with metformin, metformin plus compound C or no treatment control. **(A)** Venn diagram of H3K27ac peaks in common or unique between metformin treatment (red), no treatment (blue) or metformin plus compound C (yellow) conditions. Changes in histone modification at differentially expressed promoters for AMPK dependent **(B)** and AMPK independent **(C)** genes. **(D)** H3K27ac peak-wise hierarchical clustering heat map of 1,502 differentially enriched peaks in response to metformin treatment (adjusted P value ≤ 0.05) showing segregation into 4 groups. The z-score scale represents mean-subtracted regularized log-transformed read counts. Cluster 1 (n = 1142) includes peaks with increased H3K27ac enrichment in response to metformin that remains elevated when also treated with compound C (metformin increased, AMPK dependent). Enriched GO terms (top right) and transcription factor binding motifs (bottom right) are shown to the right of the heat map.

To identify metformin-responsive regulatory elements in a genome-wide manner, we performed ChIP-seq on primary human hepatocytes treated with the same three conditions in our RNA-seq (vehicle control, metformin, compound C and metformin) using antibodies for active (H3K27ac) and silenced (H3K27me3) histone marks (Fig 1A). For H3K27ac, we annotated 19,232 peaks in non-treated cells compared to 21,120 upon metformin treatment and 32,311 peaks in cells treated with compound C and metformin (Fig 1B; S4 Table), for a total of 34,910 distinct peaks across all conditions. Of these peaks 12,847 where shared between the non-treated and metformin treated cells, while 14,211 were unique to the compound C and metformin treatment (Fig 4A). For the H3K27me3 repressive mark, we identified 20,844 peaks in non-treated cells compared to 45,909 upon metformin treatment, and 28,105 peaks with treatment of compound C and metformin (Fig 1B; S4 Table). The higher number of peaks identified in metformin treated cells (45,909) is likely due to technical differences, as the replicates for the other conditions showed larger variation between one another.

To gain an understanding of chromatin changes in response to metformin treatment, we used our ChIP-seq data to characterize the average changes in H3K27ac and H3K27me3 around transcription start sites (TSSs) of DE genes. We observed that H3K27ac increased around the TSS of genes that were significantly upregulated by metformin, while H3K27me3 marks remained unchanged with no detectable signal in either condition (Fig 4B). However, among TSSs of significantly downregulated genes, H3K27ac coverage remained unchanged after treatment with metformin and H3K27me3 remained around zero in both conditions. We also carried out an enrichment analysis between non-treated and metformin treated H3K27me3 peaks. We did not identify any differentially enriched peaks, further suggesting that H3K27me3 marks are not responsive to metformin treatment, and differences in peak numbers may be due to ChIP efficiency. Combined, these analyses suggest that H3K27ac undergoes more dynamic changes in response to metformin than H3K27me3 (Fig 4C).

We next generated AMPK-dependent and AMPK-independent clusters of H3K27ac peak regions by comparing these three conditions (Fig 4D). Cluster one (n = 1142) contained regions that showed increased H3K27ac marks upon treatment of metformin alone, which is then reduced with combined treatment of compound C. Similar to the RNA-seq analysis, these regions were termed metformin increased, AMPK-dependent. Using the Genomic Regions Enrichment of Annotations Tool (GREAT [32]; Fig 4D; S5 Table) we observed enrichment of peak regions nearby genes belonging to several GO categories, including negative regulation of protein phosphorylation and regulation of fatty acid metabolic process. Additionally, peak regions belonging to this cluster where enriched for *ATF3* binding motifs (Fig 4D). Cluster four (n = 70) contains regions where H3K27ac histone modification decreases with metformin treatment, but returns to untreated levels with the combined treatment of compound C and metformin.

We also identified AMPK-independent H3K27ac peak region clusters. As compound C is also thought to have off-target effects [31], we only considered peaks whose expression changed due to metformin treatment for this assay. Cluster two (n = 87) contains peak regions whose H3K27ac enrichment increases with metformin treatment that remain elevated with the combined treatment of compound C and metformin (S4 Table). We termed these regions as metformin increased, AMPK-independent. Cluster three (n = 203) contain regions with H3K27ac histone modifications that decrease with metformin treatment and that remain low with the combined treatment of compound C and metformin. These regions were termed metformin decreased, AMPK-independent and were associated with several metabolic pathways and xenobiotic stimulus (S5 Table).

### Functional validation of putative metformin induced promoters and enhancers

**Fig 5:**
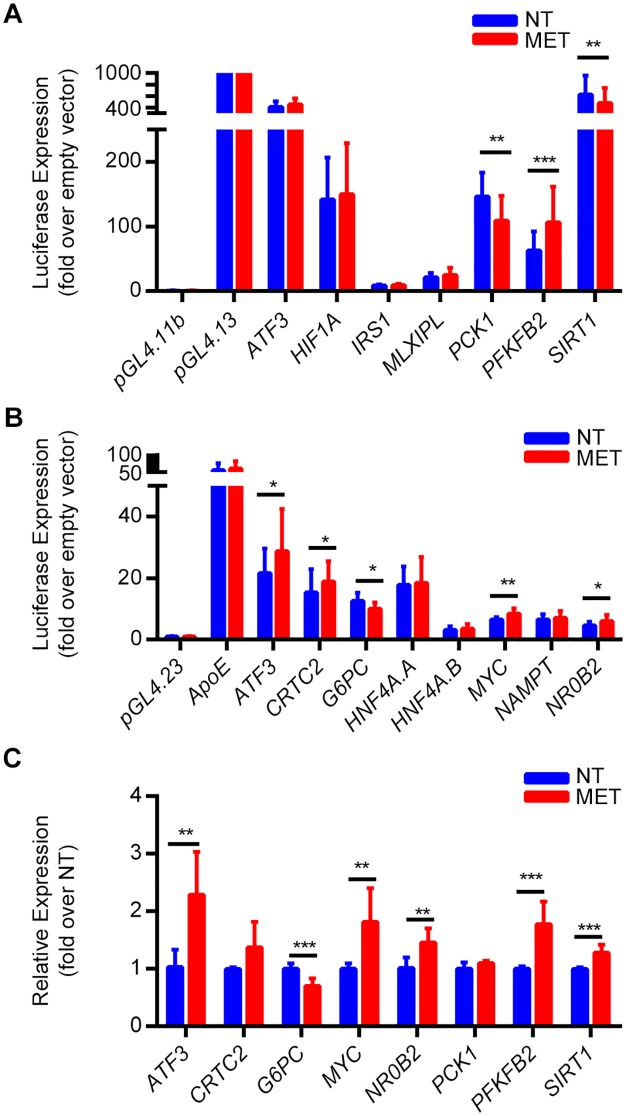
Functional validation of putative metformin induced promoters and enhancers. **(A)** Luciferase results for sequences that showed significant promoter activity when tested in Huh-7 liver cells treated with 2.5 mM metformin for 8 hours or vehicle control. An empty vector, pGL4.11b, was used as a negative control and the pGL4.13, which contains a strong SV40 promoter and enhancer, was used as positive control. **(B)** Luciferase results for sequences that were tested for enhancer activity in Huh-7 liver cells treated with 2.5 mM metformin for 8 hours or vehicle control. An empty vector pGL4.23 was used as a negative control and the ApoE liver enhancer [33] was used as positive control. **(C)** Gene expression levels in Huh-7 cells treated with vehicle control or 2.5 mM metformin for 8 hours as determined by qPCR. *P < 0.05; **P < 0.01; ***P < 0.001 (unpaired t-test).

To functionally validate our ChIP-seq results, we tested putative promoter and enhancer sequences that had a H3K27ac peak upon metformin treatment. We first analyzed the relative H3K27ac peak intensity between non-treated and metformin treated cells and identified 1,517 differentially enriched peaks (S6 Table). For promoter assays, we selected ten promoters of genes known to be involved in metformin response, several of which were enriched for H3K27ac upon metformin treatment (S7 Table). These sequences were cloned into a promoter assay vector (pGL4.11b; Promega) and tested for their promoter activity in Huh-7 liver cells treated with 2.5 mM metformin for 8 hours or vehicle control. Among these promoters, seven exhibited promoter activity, and three were found to have differential activity upon metformin-treatment (Fig 5A; S7 Table). These three include the *PFKFB2* promoter that was upregulated, and the *PCK1* and *SIRT1* promoters that were downregulated upon metformin treatment. We performed quantitative PCR (qPCR) for these genes in Huh-7 cells and confirmed that *PFKFB2* was increased by metformin. However, *PCK1* did not show a significant change and *SIRT1* was actually increased in these assays (Fig 5C). Combined, these results suggest that potentially additional regulatory elements, such as enhancers, could control metformin response.

We next set out to test putative enhancer sequences for their activity using a similar luciferase reporter assay. We selected fourteen putative enhancer sequences many of which had H3K27ac metformin enriched peaks and that reside near genes whose expression was induced by metformin or genes known to be associated with metformin response based on the literature (S7 Table). These sequences were cloned into an enhancer assay vector (pGL4.23; Promega), which contains a minimal promoter followed by a luciferase reporter gene. As a positive control, we used the *ApoE* liver enhancer [33], whose activity should not be enhanced by metformin, and the pGL4.23 empty vector as a negative control. All constructs were tested for their enhancer activity in Huh-7 cells treated with 2.5 mM metformin for 8 hour or vehicle control, similar to the promoter assays. Out of the 14 assayed sequences, 8 showed significant enhancer activity and 4 were significantly induced upon metformin treatment (Fig 5B; S7 Table). These 4 metformin induced sequences reside near *ATF3*, *CRTC2*, *NR0B2* and *MYC* genes. In addition, a sequence near *G6PC* was significantly repressed upon metformin treatment. We performed qPCR for these genes in Huh-7 metformin treated and untreated cells and found that *ATF3*, *NR0B2* and *MYC* were upregulated and *G6PC* was downregulated by metformin (Fig 5C). For the *CRTC2*, we did not observe a significant upregulation, but did see a trend for metformin induction (Fig 5C). Our qPCR results overall agree with our enhancer assays, suggesting that these enhancers and genes could be regulated by metformin.

### Differential enhancer activity of a metformin treatment associated haplotype in the *ATM* locus

**Fig 6:**
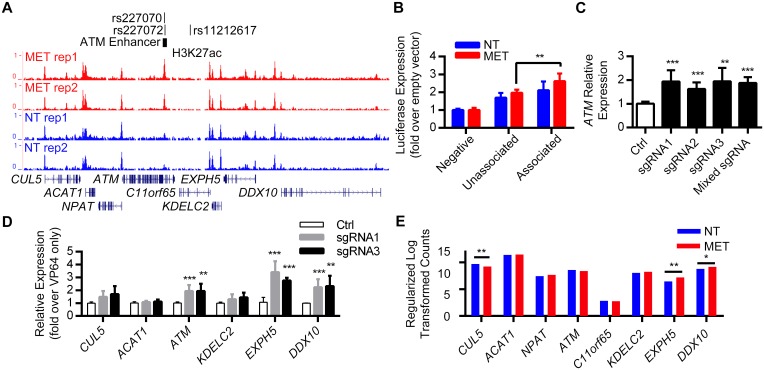
Differential enhancer activity of a metformin treatment associated haplotype in the *ATM* locus. **(A)** Integrative genomic viewer snapshot showing the genomic location of the H3K27ac metformin enriched peak along with the various SNPs. **(B)** The treatment response associated haplotype shows significantly higher enhancer activity following metformin treatment compared to the unassociated haplotype (p = 0.007; unpaired t-test). **(C)** *ATM* expression as assessed by qPCR in Huh-7 cells transfected with dCas9-VP64 and three sgRNAs targeting the *ATM* intronic enhancer. Data are expressed as fold increase relative to the control sample which was only transfected with the dCas9-VP64 vector. **(D)** Expression analysis of other genes in the locus following CRISPRa with sgRNAs 1 and 3 as assessed by qPCR in Huh-7 cells and compared to cells transfected only with dCas9-VP64. *P < 0.05; **P < 0.01; ***P < 0.001 (unpaired t-test). **(E)** RNA-seq expression values of genes near the *ATM* intronic enhancer displayed as the regularized log transformed counts derived from DESeq2. Genes differentially expressed in response to metformin are noted by asterisk (*adjusted P ≤ 0.05, **adjusted P ≤ 1e-4).

A noncoding SNP, rs11212617, in the *ATM* locus was previously reported to be associated with metformin treatment success in a GWAS for glycemic response to metformin [25]. To test for potential gene regulatory elements in this locus, we analyzed our ChIP-seq data for metformin enriched peaks in this locus. We identified a metformin H3K27ac enriched peak in this region within an intron of the *ATM* gene that contains SNPs rs277070 and rs277072 that are in LD with rs11212617 [R^2^>0.95 in the Caucasian (CEU) population] (Fig 6A). We cloned both the unassociated and associated haplotype of this intronic peak into our enhancer assay vector and carried out similar luciferase reporter assays in Huh-7 cells as described above. Both sequences showed significant enhancer activity, which was increased upon metformin response. However, the treatment response associated haplotype showed significantly higher enhancer activity upon metformin response compared to the unassociated haplotype (Fig 6B). Our findings suggest that the haplotype that is in LD with the GWAS lead SNP rs11212617 increases enhancer activity and could lead to elevated expression of its target gene/s.

To identify potential target genes for this enhancer, we analyzed eQTL data and also took advantage of CRISPR/Cas9 activation technology (CRISPRa; [34]) to increase the activity of this enhancer and measure changes in expression levels of the nearby genes. eQTL analysis of SNPs rs277070 and rs277072 (see methods) found significant associations with increased *ATM* mRNA expression for both SNPs (S5 Fig; S8 Table). For CRISPRa, we infected Huh-7 cells with a nuclease-deficient Cas9 (dCas9) fused to the VP64 transcriptional activator along with three different single guided RNA (sgRNA) targeting the enhancer. We generated mRNA and measured the expression levels of *ATM* in this region compared to cells with only dCas9-VP64. We found that *ATM* expression is significantly increased by about 2 fold by all three sgRNAs and when all 3 sgRNAs were infected together (Fig 6C), with sgRNA1 and sgRNA3 providing the highest activation results. We then measured the expression levels of additional genes in this locus [Cullin 5 (*CUL5*), Acetyl-CoA Acetyltransferase 1 (*ACAT1*), Nuclear Protein, Ataxia-Telangiectasia Locus (*NPAT*), Chromosome 11 Open Reading Frame 65 (*C11orf65*), DEAD (Asp-Glu-Ala-Asp) Box Polypeptide 10 (*DDX10*)], finding that not only *ATM*, but also *EXPH5* (around 4 fold) and *DDX10* (around 2.5 fold) expression was significantly increased by both sgRNA1 and sgRNA3 (Fig 6D). Interestingly, *EXPH5* and *DDX10* (but not *ATM*) were upregulated by metformin in our RNA-seq data (Fig 6E; S1 Table), which is consistent with the possible activation of these two genes by this enhancer. Combined, these results suggest that this enhancer could be targeting either *ATM*, *EXPH5* and/or *DDX10*.

### *ATF3* expression is dependent upon AMPK activation

**Fig 7:**
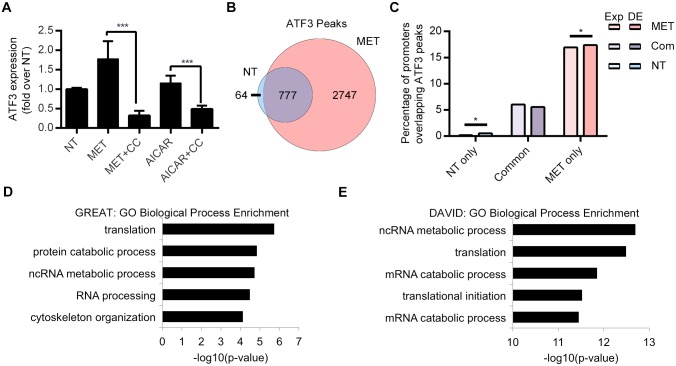
ATF3 ChIP-seq results. **(A)** *ATF3* expression analysis in Huh-7 cells treated with either vehicle control, metformin, metformin + compound C, AICAR or AICAR + compound C. **(B)** Venn diagram of ATF3 peaks in common (n = 777) or unique to metformin treatment (n = 2747; red), no treatment (n = 65; blue). **(C)** Enrichment of ATF3 peaks overlapping promoters of differentially expressed (DE) genes compared to a background of all genes with sufficient read counts to be tested for differential expression (denoted Exp). Peaks unique to non-treated (NT) and metformin treated (MET) conditions showed a significant enrichment based on a random permutation test (n = 2000, *P < 0.02), while no such enrichment was found for peaks in common (abbreviated Com) between these conditions. Enriched GO terms for all metformin unique ATF3 peaks as found by **(D)** GREAT and their nearest gene by **(E)** DAVID.

*ATF3* was the top differentially expressed AMPK-dependent gene in our metformin treatment (Fig 2B). *ATF3* is a member of the ATF/cyclic adenosine monophosphate (cAMP) responsive element-binding protein family of transcription factors, a stress response gene that both represses and activates genes[35,36]. *ATF3* is known to be involved in metformin response in macrophages [37], but its role in metformin response in the liver is not well characterized. To better characterize the role of ATF3 in hepatic metformin response we carried out various assays including ATF3 ChIP-seq, siRNA and pathway analyses. We first wanted to validate that *ATF3* was indeed an AMPK-dependent gene, as observed in our RNA-seq results. We thus treated Huh-7 cells with metformin, AICAR (an AMPK activator), or compound C (an AMPK inhibitor). We found that *ATF3* expression was induced by both metformin and AICAR treatment, but not with compound C (Fig 7A), further confirming that *ATF3* expression is dependent on AMPK, and that AMPK activation is required for *ATF3* induction.

### ATF3 ChIP-seq

To obtain a genome-wide view of ATF3 function following metformin response, we carried out ATF3 ChIP-seq on primary human hepatocytes from the same individual used in our previous RNA-seq and ChIP-seq assays treated with 2.5mM metformin for 8 hours and compared them to vehicle control, similar to previous ChIP-seq experiments. Since *ATF3* is an AMPK-dependent gene, we did not carry out an additional compound C + metformin experiment. We observed a massive recruitment of ATF3 binding across the genome following metformin treatment. ATF3-bound DNA fragments clustered into 842 discrete peaks in non-treated cells compared to 3,535 upon metformin treatment (Fig 1A, S4 Table), with 777 overlapping in both datasets (Fig 7B). Analysis of ATF3 metformin-treated unique peaks overlapping gene promoters found an enrichment of peaks occurring in DE genes as compared to all genes (Fig 7C), including *KLF6*, *NR0B2*, *DUSP1*, *EIF2AK3*, *IRS1*, *NAMPT* all known metformin-associated genes. We also observed a peak overlapping the *ATF3* promoter suggesting that it autoregulates itself. Further analysis of the ATF3 peaks from metformin treated cells using both the Database for Annotation, Visualization and Integrated Discovery (DAVID) [38] and GREAT [32] found them to be enriched near genes involved in protein translation, noncoding RNA metabolic processes, RNA processing, cytoskeleton organization, transcription activation, ATP binding and many others (Fig 7D and 7E; S9 Table). In addition, we performed pathway analysis using IPA of genes found near ATF3-H3K27ac metformin peaks and found an enrichment for the top “EIF2 signaling” canonical pathway that is involved in protein translation (S6 Fig; S10 Table).

### Knockdown assays associate *ATF3* with an important role in metformin induced gluconeogenesis

**Fig 8:**
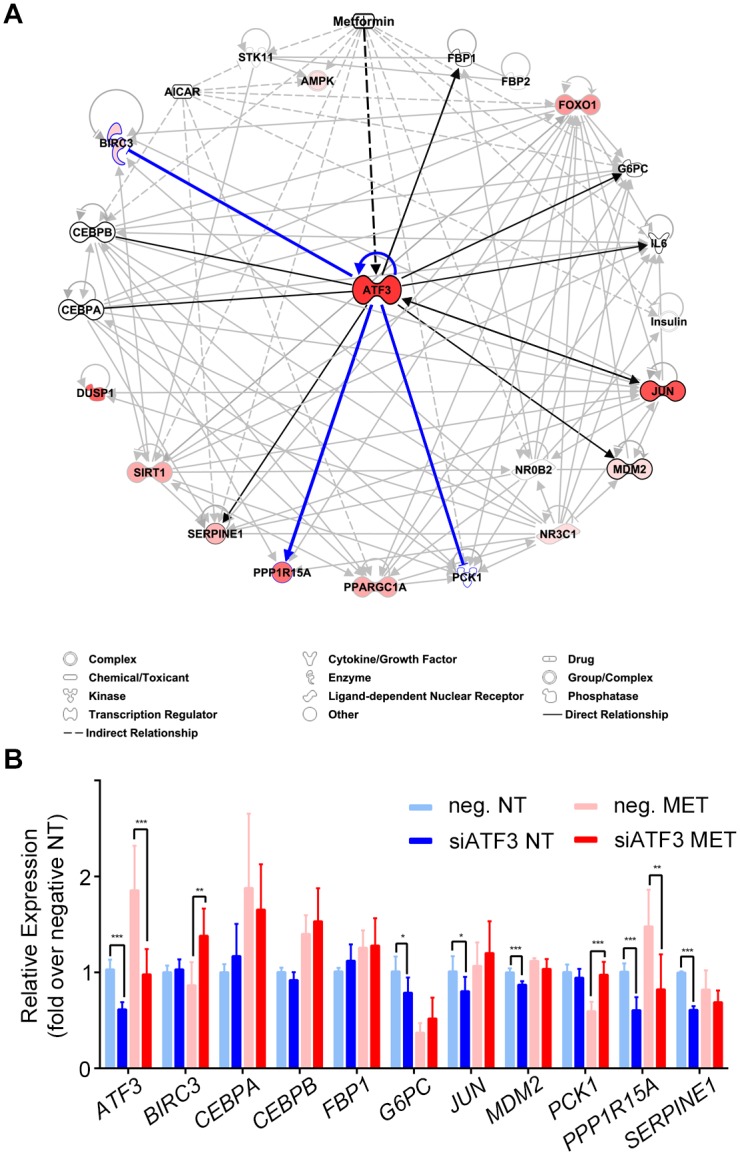
*ATF3* pathway and siRNA analyses. **(A)** *ATF3* pathway analysis showing its interaction with metformin, AICAR and genes related to gluconeogenesis. Upregulated genes, as determined by RNA-seq, are colored in red. Black and blue lines show the genes assayed following *ATF3* siRNA, with blue lines indicating genes that showed significant differences in expression levels in the metformin treatment following *ATF3* knockdown. **(B)** Gene expression levels following *ATF3* knockdown in Huh-7 cells treated with either negative control or *ATF3* siRNA and with either 2.5 mM metformin or vehicle control for 8 hours. *P < 0.05; **P < 0.01; ***P < 0.001 (unpaired t-test).

We next set out to better characterize the functional role of *ATF3* following metformin treatment. Using IPA, we generated an ATF3 metformin response pathway centered on its interaction with metformin, AICAR and genes related to gluconeogenesis (Fig 8A). We then selected *ATF3* regulated genes predicted by this pathway to be analyzed following *ATF3* siRNA knockdown in Huh-7 cells. We first validated that this siRNA can knockdown *ATF3*, observing a 40% and 48% *ATF3* mRNA reduction in non-treated and metformin treated cells respectively (Fig 8B). Next, we knocked down *ATF3* in Huh-7 cells that were then treated with vehicle control or metformin, and performed qPCR on selected genes that were predicted by the *ATF3* pathway (Fig 8A). In the basal condition, we observed significant downregulation following *ATF3* knockdown for *G6PC*, *JUN*, *MDM2*, *PPP1R15A* and *SERPINE* when compared to the siRNA control (Fig 8B). Upon metformin treatment, we observed a significant downregulation due to *ATF3* knockdown for *PPP1R15A* and a significant upregulation of *BIRC3* and *PCK1* when compared to the negative siRNA control (Fig 8B). These results suggest that *ATF3* activates *PPP1R15A* and represses *BIRC3* and *PCK1* upon metformin activation (Fig 8A). *PPP1R15A* codes for the protein phosphatase 1, regulatory subunit 15A, which recruits the serine/threonine-protein phosphatase PP1 to dephosphorylate the translation initiation factor eIF-2A/EIF2S1. Interestingly, reducing the hepatic eIF2α signaling pathway in mice was shown to lead to reduced hepatic glucose production through reduced hepatic gluconeogenic gene expression [39]. *BIRC3* codes for the protein baculoviral IAP repeat containing 3, and its expression was associated with the survival of insulin-secreting human liver cell line, which restored normoglycemia when transplanted into diabetic immunoincompetent mice [40]. Furthermore, *PCK1* encodes the phosphoenolpyruvate carboxykinase 1 (PEPCK) enzyme, which catalyzes the conversion of oxaloacetate to phosphoenolpyruvate and carbon dioxide and is considered a key pathway for hepatic gluconeogenesis [41]. In combination, our findings suggest that *ATF3* responds to metformin and could be involved in gluconeogenesis repression.

## Discussion

We have systematically characterized metformin hepatic response using RNA-seq and ChIP-seq on primary human hepatocytes treated with vehicle control, metformin, and compound C and metformin. We identified AMPK-dependent and AMPK-independent clusters from 1,906 differentially expressed (DE) genes. To identify metformin-associated regulatory elements, we carried out ChIP-seq for H3K27ac, a known active mark [28], and H3K27me3 a repression mark [28]. To functionally validate our results, we carried out functional reporter assays in Huh-7 cells with similar treatments finding several promoter and enhancer sequences to be modulated by metformin. Moreover, enhancer assays of a metformin increased H3K27ac ChIP-seq peak in an *ATM* intron that contained SNPs in LD with the metformin treatment response GWAS lead SNP (rs11212617) showed increased enhancer activity for the associated haplotype. eQTL liver analysis suggests that SNPs within this enhancer are associated with increased *ATM* expression. Using CRISPRa, we showed that this enhancer could be regulating either *ATM*, *EXPH5* and/or *DDX10*. Finally, using ChIP-seq and siRNA, we characterized the metformin-associated function of *ATF3*, the top upregulated AMPK-dependent gene, finding it to have an important role in gluconeogenesis repression.

Our RNA-seq analyses identified novel transcription factors associated with metformin response. For example, krüppel-like factor 6 (*KLF6*) was found among the upregulated AMPK-dependent genes. *KLF6* was implicated as a novel regulator of hepatic glucose and lipid metabolism in non-alcoholic fatty liver disease, characterized by dysregulated glucose homeostasis [42]. Conversely, ajuba LIM protein (*AJUBA*) was found among the downregulated AMPK-independent genes. It is thought to indirectly affect the activity of Protein Kinase Cζ [43], which is required for LKB1 phosphorylation and, thus, AMPK activation [44]. Notably, our IPA upstream regulator analysis for DE genes (Fig 3) support that metformin affects the expression of many novel transcriptional regulators related to gluconeogenesis. *KLF6* isoforms were also found to be involved in tumor progression [45], and the activity of AJUBA was found to promote cancer growth [46]. Moreover, network analyses showed that *CDKN1A*, *ESR1*, *MAX*, *MYC*, *PPARGC1A*, and *SP1* play important roles in the antidiabetic and anticancer effects of metformin [47]. Notably, these genes were also found to be DE in our RNA-seq (S1 Table), and these findings support metformin as an emerging candidate for cancer therapy [5,6].

RNA-seq analyses also identified novel protein coding genes associated with metformin response. For example, dual specificity phosphatase 10 (*DUSP10*) was found among the upregulated AMPK-dependent genes (Fig 2B). This gene negatively regulates members of the mitogen-activated protein kinases (MAPK) superfamily, some of them proposed as tumor suppressors [48]. DUSP10 preferentially dephosphorylates p38 MAPK [49]. Growth differentiation factor 15 (*GDF15*) was the top upregulated AMPK-independent gene (Fig 2B) and its overexpression is known to result in improved insulin sensitivity [50]. The transporter solute carrier family 19, member 3 (*SLC19A3*) was also an upregulated AMPK-dependent gene (S1 Table), and is known to play a role in the intestinal absorption and tissue distribution of metformin [51]. These findings further support that metformin induces protein coding genes which play a role in cancer and T2D.

Our promoter assays identified several functional promoters, but only a few were differentially regulated by metformin. The promoter for *PFKFB2* was induced by metformin, and showed increased expression by metformin both in our qPCR (Fig 5C) and RNA-seq (S1 Table). This gene is involved in both the synthesis and degradation of fructose-2,6-bisphosphate, a regulatory molecule that controls glycolysis. However, we found discordant findings with the literature for other promoter assays. For example, *PCK1* codes for the PEPCK enzyme, a main control point for hepatic gluconeogenesis, which catalyzes the conversion of oxaloacetate to phosphoenolpyruvate and carbon dioxide [41]. *PCK1* was upregulated by metformin in our RNA-seq (S1 Table), but the promoter for *PCK1* was repressed by metformin, and *PCK1* expression was decreased by metformin in Huh-7 cells (Fig 5C). Finally, *SIRT1* codes for the sirtuin 1 protein, which leads to the suppression of gluconeogenic expression. Metformin increases SIRT1 activity through activation of AMPK [52]. *SIRT1* showed increased expression by metformin both in our qPCR (Fig 5C) and RNA-seq (S1 Table). However, the promoter for *SIRT1* was repressed by metformin. These results could imply that although promoters can respond to metformin they may not be the only responders, suggesting a role for other metformin-responsive elements, such as enhancers.

Enhancers have been identified as potential determinants of drug response [53,54]. Here, we used ChIP-seq for H3K27ac to identify metformin-responsive associated enhancers and found putative enhancer sequences near genes related to metformin action on gluconeogenesis. Notably, an enhancer sequence near *G6PC*, which has a major role in gluconeogenesis [12], was repressed by metformin (Fig 5B). Moreover, we found an enhancer that was induced by metformin near *NR0B2*, which codes for the nuclear receptor SHP, a transcriptional repressor that inhibits CREB-dependent hepatic gluconeogenic expression via direct interaction and competition with coactivators [12,15]. *NR0B2* was also upregulated by metformin in our RNA-seq (S1 Table) and qPCR (Fig 5C). We also found a metformin inducible enhancer near *CRTC2*, a transcription coactivator that is a key regulator of fasting glucose metabolism [55]. It is worth noting that these sequences would not have been identified by conventional ChIP studies conducted in physiological conditions, nor would they be validated in functional assays without drug treatment, highlighting the need to carry out these drug-induced studies. Together, our result suggests that ChIP-seq datasets are dependent on the environmental conditions in which they were performed, and that there are likely many enhancers which are only observed following a specific stimulus.

We also followed up on an *ATM* intronic enhancer that encompassed SNPs in LD with a GWAS SNP that was associated with metformin treatment success [25]. Enhancer assays showed increased enhancer activity upon metformin response for the associated haplotype (Fig 6B), suggesting that it could increase enhancer activity and elevate the expression of its target gene/s. eQTL analysis showed that the SNPs within this enhancer are associated with increased *ATM* expression. Next, we used CRISPRa to further identify the target gene/s. Although *ATM* expression was increased by three sgRNA targeting this enhancer region, this gene may not be the only gene regulated by this enhancer region. *EXPH5* and *DDX10* expression were also significantly increased. Interestingly, *EXPH5* and *DDX10*, but not *ATM*, were upregulated by metformin in our RNA-seq, which are consistent with the possible metformin induction of these two genes by this enhancer. The SNP rs11212617 falls within a large block of LD including *CUL5*, *ACAT1*, *NPAT*, *ATM*, *C11orf65*, *KDELC2*, *EXPH5* [25]. Interestingly, we noted from GRASP v2.0 database [56] that SNPs near *DDX10* are associated with fasting insulin and HOMA-IR (p<3x10^-5^) [57], and SNPs near *EXPH5* are associated with fasting glucose (p<3x10^-3^) [58]. However, to our knowledge, no previous study has related *EXPH5* or *DDX10* to metformin response.

ATF3 is a transcription factor which can act as either a transcriptional activator or repressor depending on the cell type and stimulus [35,36]. Metformin is known to cause anti-inflammatory effects, and it was suggested that metformin exhibits anti-inflammatory action in macrophages at least in part via pathways involving AMPK activation and ATF3 induction [37]. To our knowledge, our study is the first to show a role for *ATF3* in hepatic metformin response. *ATF3* was the top upregulated gene in our RNA-seq in human hepatocytes treated with metformin (Fig 2B), which was further confirmed by qPCR in Huh-7 cells (Fig 5C). *ATF3* activity was shown to be AMPK-dependent in our RNA-seq, which we further validated by using AICAR (an AMPK activator) and compound C (an AMPK inhibitor), increasing *ATF3* expression only in the AICAR condition (Fig 7A). Taken together, these findings suggest that *ATF3* induction is dependent on metformin-induced AMPK activation. For ChIP-seq, it is worth noting that we used the antibody against both the shorter and the longer ATF3 isoform, which have stimulatory and repression roles on transcription, respectively [35]. Our analysis of metformin increased H3K27ac peaks showed enrichment for *ATF3* binding motifs (Fig 4D). Interestingly, ATF3 was also recently shown to bind genomic sites enriched with EP300 and H3K27ac for transcriptional regulation [59]. Moreover, analysis of ATF3 marks unique to either the non-treated or metformin treated conditions were found to be overrepresented in differentially expressed H3K27ac peaks when compared to all H3K27ac peaks. Conversely, shared ATF3 peaks between both conditions (non-treated and metformin treated) were significantly underrepresented in these differentially expressed H3K27ac peaks (S7 Fig).

To better characterize the functional role of ATF3 upon metformin treatment, we used siRNA to knockdown *ATF3*. We found a significant downregulation for *PPP1R15A* and a significant upregulation of *BIRC3* and *PCK1* when compared to the negative siRNA control (Fig 8B), which suggest that *ATF3* activates *PPP1R15A* and represses *BIRC3* and *PCK1* upon metformin activation (Fig 8A). Notably, *PPP1R15A* and *BIRC3* were found among the upregulated AMPK-dependent genes in our RNA-seq. *PPP1R15A* is known to dephosphorylate the translation initiation factor eIF-2A/EIF2S1, which is thought to lead to reduced hepatic glucose [39]. *BIRC3* is associated with the survival of insulin-secreting human liver cells, which were shown to restore normoglycemia when transplanted into diabetic immunoincompetent mice [40]. Interestingly, metformin was shown to inhibit the expression of *Birc3*, and to upregulate *Ppp1R15a* expression in mice [60]. Furthermore, *PCK1* encodes the PEPCK enzyme, which is considered a key pathway for hepatic gluconeogenesis [41]. Notably, ATF3 was shown to repress the *PEPCK* promoter, providing a potential mechanistic explanation for the reduced *PEPCK* expression in transgenic mice [61]. To our knowledge, our study is the first to show that *ATF3* represses *PCK1* upon metformin activation in human hepatocytes. Taken together, these findings suggest that *PCK1* is repressed and further connect *ATF3* in gluconeogenesis repression.

It is worth noting that there are several caveats to our study. Our assays were done in primary human hepatocytes, and not tissues, treated with 2.5 mM metformin in order to observe significant metformin response, which is higher than physiological conditions used to treat metformin in patients. Our cells also do not mimic as well hepatocytes in their *in vivo* environment compared to more complex culturing conditions such as micropatterned cocultures [62] or a collagen sandwich system [63]. Due to the large costs and limited hepatocyte material, the cells were only treated in a single concentration of compound C (40 μM) which was previously shown to be effective in suppressing metformin-stimulated AMPK phosphorylation and glucose production [11,64]. Our ChIP-seq experiments only analyzed hepatocytes from a single donor at a single time point (8 hours post treatment), and there could be many genes and enhancers that play a role in different time-points. To test regulatory sequences in reporter assays, we used the pGL4.23 vector, which is a commonly used enhancer assay vector, but possesses a very short TATA-box containing minimal promoter that may not be compatible with all of the enhancer sequences tested. In addition, because of the difficulties in transfecting primary cells and due to having a limited amount of primary hepatocytes from the same donor, our reporter assays were carried out in Huh-7 liver cells, which due to being established cell lines, could result in discordant findings. Nonetheless, our study provides a genome-wide understanding of metformin response in human primary hepatocytes, highlighting important sequences that could be associated with the variability in response to metformin, and enabling the identification and prioritization of novel candidates for T2D treatment.

## Methods

### Primary human hepatocytes

Cryopreserved human hepatocytes from a 19 year old Caucasian male donor with no history of medications (Lot Hu8080, Life Technologies) as were used in [65] were thawed in Cryopreserved Hepatocyte Recovery Medium (Cat#CM7000, Life Technologies) to enhance the recovery of viable hepatocytes while removing cryoprotectant after cell cryopreservation. They were then cultured in Cryopreserved Hepatocyte Plating Medium (Cat#CM9000, Life Technologies) on 6-well collagen-coated plates (Life Technologies) for optimal attachment and monolayer formation. After 6 hours, the plating medium was swapped with maintenance media, consisting of phenol-free Williams E media containing culture incubation supplements (Cat#CM4000, Life Technologies). After 24 hours, the maintenance media was replaced by vehicle control (only media), 2.5 mM Metformin (Sigma-Aldrich, Cat#PHR1084) or 2.5mM metformin and 40μM Compound C (Sigma-Aldrich, Cat#P5499) for 8 hours. Dexamethasone, which is included separately with the culture incubation supplements, was not added to the media, in order to prevent any possible unknown metformin-responsive gene activation [65].

### Western blot of p-AMPK for metformin induction

Primary human hepatocytes were treated with vehicle or metformin (0.5 mM, 2.5 mM or 10 mM) for 4 or 8 hours in duplicate (S1 Fig). The cells were lysed using RIPA buffer (Sigma) with protease inhibitors (Roche Applied Science). After centrifugation for 20 minutes at 16,100 *g* at 4°C, the supernatants were removed for determination of protein content and separated on NuPAGE Novex 4–12% Bis-Tris Gel (Life Technologies). 40 micrograms of proteins from the supernatant were separated and transferred to PVDF membrane. The membranes were blocked overnight at 4°C with Tris-buffered saline with 0.05% Tween 20 and 5% nonfat milk. Immunoblotting was performed following standard procedures, and the signals were detected by chemiluminescence reagents (GE healthcare, NJ). Primary antibodies were directed against: AMPKα (1:1000), AMPKα phosphorylated at Thr172 (1:500) and β-actin (1:2000) (Cell Signaling Technology, Danvers, MA). ImageJ (http://rsb.info.nih.gov/ij/index.html) was used to quantify.

### RNA-seq

Cultured primary human hepatocytes from the same cells/donor were treated with metformin, metformin + compound C or no treatment for 8 hours in triplicate. The cells were then washed with PBS, and lysed directly with Buffer RLT Plus. Total RNA was extracted using the AllPrep DNA/RNA Mini Kit (Cat#80204; Qiagen). Sequencing was carried out on an Illumina HiSeq 2000 to a depth of at least 30 million reads. The resulting reads were aligned to the human genome (hg19) using HISAT 0.1.5-beta [66]. Analysis for differential expression across the three conditions was performed by first obtaining read counts for each gene using HTSeq [67] and tests of differential expression were carried out using DESeq2 v 1.10.1 [68]. After differential expression testing, AMPK dependent or independent genes were identified through clustering analysis on all genes with adjusted p-values (FDR) <0.05 between the metformin treated and non-treated conditions using the R package hclust and displayed in a heatmap.

### Ingenuity pathway analysis

Ensembl Gene IDs and the log2FoldChange for the differentially expressed (DE) genes from our RNA-seq were uploaded into QIAGEN’s Ingenuity Pathway Analysis (IPA, QIAGEN Redwood City, http://www.ingenuity.com/). We used the following IPA core analysis settings: general settings, reference set = Ingenuity knowledge base (genes only), relationships to consider = direct and indirect relationships; data sources = all; confidence = experimentally observed only; species = all. We reviewed Ingenuity canonical pathways to study known biological pathways and processes of interest that were enriched in our DE genes. We also used the Ingenuity upstream analysis, which predicts the functional status of upstream regulators, such as transcription factors, kinases, and growth factors based on known downstream targets, i.e., the input set of the DE genes.

### ChIP-seq

Twelve million cells per immunoprecipitation that were cultured separately from the RNA-seq experiments were fixed with 1% formaldehyde for 5 min and quenched with 0.125 M glycine. The remainder of the ChIP protocol was carried out using the LowCell# ChIP kit (Cat#C01010072, Diagenode) according to the manufacturer’s protocol with modifications, as follows. Chromatin was isolated by adding lysis buffer, and lysates were sonicated by Covaris S2200 ultrasonicator (Covaris, Inc.) and the DNA sheared to an average length of 300–500 bp. An aliquot of chromatin (30 ug) was pre-cleared with Protein A-coated magnetic beads (Cat# kch-802-660, Diagenode). We then used the following concentration of antibodies: 9 ug of antibody against H3K27ac (Cat#ab4729, Abcam) 9 ug antibody against H3K27me3 (Cat#ab6002, Abcam) and 5 ug of antibody against ATF3 (C-19) (Cat#sc-188, Santa Cruz). Experiments were carried out in replicates and a chromatin input sample was used as a reference for peak calling. We only worked with those peaks in both replicates for all ChIP-seq markers. Two biological replicates from the same cells/donor were carried out for each antibody. Complexes were washed, eluted from the beads with SDS buffer, and subjected to RNase and proteinase K treatment. Crosslinks were reversed by incubation overnight at 65°C, and ChIP DNA along with an aliquot of genomic DNA (Input) were purified using IPure kit (Cat#C03010011, Diagenode).

Sequencing libraries were generated using the ThruPLEX DNA-seq Kit (Rubicon Genomics, Inc) for 11 cycles. The resulting amplified DNAs were purified by Agencourt AMPure XP (Protocol 000387v001, Agencourt Bioscience), quantified by an Agilent 2100 Bioanalyzer (Agilent Technologies), and sequenced on an Illumina HiSeq 2000. Sequencing reads were mapped to the genome using bowtie allowing 1 mismatch per read-alignment and only uniquely aligned reads (-v 1 -m 1). Peaks were called against input using MACS2 version 1.4[69] for H3K27ac, H3K27me3 and ATF3. Reliable peaks were identified using the ENCODE IDR (Irreproducible Discovery Rate) pipeline, which establishes a p-value cutoff to accept peak calls for each condition. For differential peak intensity analysis, peaks across conditions were then merged for H3K27ac and H3K27me3, respectively, and reads coverage obtained using HTSeq[67]. Peaks differentially enriched for H3K27ac or H3K27me3 histone marks were then identified using DESeq2[68]. As with the RNA-seq analysis, AMPK dependent or independent peak regions were identified through clustering analysis on all genes with adjusted p-values (FDR) <0.05 between the metformin treated and non-treated conditions using the R package hclust and displayed in a heatmap. For ATF3, peaks unique to metformin treated or non-treated hepatocytes were obtained by first merging reliable peaks between both conditions with the bedtools function [70] mergeBed, then overlapping these merged peaks using intersectBed.

### Luciferase reporter assays

Promoter sequences were selected based on overlapping a metformin treatment H3K27ac ChIP-seq peak. To select putative enhancer sequences, we searched for regions across the genome which overlap metformin treated H3K27ac or ATF3 peaks and that reside near genes whose expression was induced by metformin or known to be associated with metformin response based on the literature. In addition, we identified a metformin increased H3K27ac peak in an intron of *ATM* that has SNPs in linkage disequilibrium with a metformin treatment response GWAS lead SNP (rs11212617)[25]. Using HaploReg v2 (http://compbio.mit.edu/HaploReg)[71] on data from the 1000 Genomes (http://1000genomes.org, on March 2014), we identified a larger set of SNPs in LD with the GWAS lead SNP rs11212617 (R^2^>0.95) in the HapMap Caucasian (CEU) population, including the SNPs rs277072 and rs277073.

Selected promoter regions were amplified from human genomic DNA (Roche) using oligonucleotides designed in Primer3 with 16 bp overhangs (5′- CCCGGGCTCGAGATCT-3′ and 5′-CCGGATTGCCAAGCTT-3′) complementary to the sequence flanking the *Bgl*II and *Hind*III sites in the pGL4.11b vector (Promega). Primers were designed to encompass the increased H3K27ac ChIP-seq peak plus up to 500 bp of sequence on either side of the transcription start site. Candidate enhancer sequences were amplified from human genomic DNA (Roche) using oligonucleotides designed in Primer3 with 16 bp overhangs (5′-GCTCGCTAGCCTCGAG-3′ and 5′CGCCGAGGCCAGATCT-3′) complementary to the sequence flanking the *Bgl*II and *Xho*I sites in the pGL4.23 Gate A vector (Promega). The regions for the selected sequences, the nearby genes, the primers and results for the promoter and enhancer reporter assays are shown in S7 Table. PCR products were cleaned using the NucleoSpin Gel and PCR Clean-up kit (Macherey-Nagel) and cloned into *Bgl*II and *Xho*I digested pGL4.23 or *Bgl*II and *Hind*III digested pGL4.11b using the Infusion HD cloning system (Clontech) then transformed into Stellar Competent Cells (Clontech). Haplotypes were verified by Sanger sequencing using various primers for the inserted DNA.

Huh-7 human liver cells (ATCC) were maintained in D-MEM (Life Technologies) supplemented with FBS (JRS Scientific), Penicillin-Streptomycin and Glutamine (UCSF Cell Culture Facility). On the day before transfection, the cells were trypsinized, washed, and diluted to a density of two hundred thousand/ml in D-MEM. Twenty thousand cells were added to each well of a 96-well clear bottom tissue culture plate (Falcon) containing the transfection mixture for the promoter and enhancer assay respectively. The transfection mixture consisted of 100 ng of either pGL4.23 ApoE or pGL4.13, 10 ng of pGL4.74, 100 ng of either empty pGL4.23 Gate A or pGL4.11b, 10 ul Opti-MEM, 100 ng of reporter construct, and 0.2 ul of X-tremeGene HP DNA Transfection Reagent (Roche). After 18 hours of incubation at 37 degrees Celsius, the media was replaced with 100 ul of either D-MEM supplemented with 2.5mM Metformin or D-MEM without any additional supplements. After 8 hours of incubation, the cells were washed with PBS and the promoter and enhancer assay cells were lysed with 25 ul of Passive Lysis Buffer (Promega). For both promoter and enhancers, reporter activity was measured using the Dual-Luciferase Reporter Assay System (Promega). Both assays were measured on the Synergy 2 Plate reader (BioTek Instruments, Inc).

### eQTL analyses

We used four liver eQTL datasets comprising a total number of 1,180 liver samples from individuals of European ancestry (S8 Table). Tissue procurement, gene expression analysis, genotyping and eQTL analyses have been described previously for three of the datasets [72–74]. The forth dataset was contributed by Dr. Eric Schadt (GEO:GSE9588; [73]). Genotypes were imputed to the 1000 Genome reference panel with Minimac (http://genome.sph.umich.edu/wiki/Minimac). Expression probe sequences were mapped to ENSEMBL genes and only the common genes across all datasets were included for subsequent analyses. Within each dataset, the genome-wide eQTL analysis was run with an additive genetic model including dataset specific covariates to examine *cis*-associations within a 100kb flanking window. Results from the four datasets were then combined with a modified meta test statistic which was calculated using the following approach: t_meta_ = (∑w_i_t_i_)/√(∑w_i_^2^), w = √(*n*−(#*cov*ariates)−1) where i = data sets 1–4 and n = sample size [75]. Generation of p-values was accomplished by assuming the meta test statistics were normally distributed.

### CRISPRa

Huh-7 cells were transfected using 2 μg of dCas9-VP64 vector and 2 μg of equimolar pooled or individual gRNA expression vectors mixed with X-tremeGene HP DNA Transfection Reagent (Roche). After 48 hour, cells were harvested and total RNA was extracted using the RNeasy Mini Kit (Qiagen). First-strand cDNA synthesis was performed using 1 μg total RNA as a template and SuperScript III First-Strand Synthesis System (Life Technologies) primed with 50 ng of random hexamers. Quantitive real-time PCR was performed using the Bio-Rad SsoFast EvaGreen Supermix (Bio-Rad Laboratories). Gene expression was normalized to of the *HPRT* housekeeping gene. All *S*. *pyogenes* gRNAs were annealed and cloned into U6 human sgRNA vector using NEB BstX1 and XhoI. sgRNA protospacer targets were designed using the sgRNA designer [76] (see S11 Table) and are as follow: gRNA1: TAGAGTATCTAACCCAACGT; gRNA2: GGATAATGTGAACTCATGTG; gRNA3: TTGCTCAGACTGAAACCTAG.

### AICAR and compound C ATF3 analyses

Huh-7 cells were seeded in 6-well plates at a density of 1x10^6^ cells/well. Cells were treated with 2.5 mM metformin or 1mM 5-aminoimidazolecarboxamide riboside (AICAR) (TOCRIS Bioscience) for 8 hours. For compound C, cells were first treated with 40 uM compound C (Sigma-Aldrich) for 30min, and then 2.5mM metformin or 1mM AICAR was added for 8 hour.

### ATF3 knockdown

A small interfering RNA (siRNA) against ATF3 (Life Technologies, 4392420-s1701) or negative control siRNA was transfected (30 nM each/well) into Huh-7 (5x10^5^ cells/well at 70–80% confluence) using Lipofectamine RNAiMAX transfection reagent (Life Technologies) following the manufacturer’s protocol. Briefly, 30nM of siRNA per well of a 6-well plate was diluted into 200 μl of OptiMEM (Invitrogen), and 12 μl of RNAiMax was diluted in 200 μl of Opti-MEM. The siRNA and RNAiMax were then combined into the same tube and incubated at room temperature for 20 minutes and then added to the cells. After a 6 hour incubation, the medium containing the complexes was replaced with 2 ml of standard DMEM media and cultured at 37°C. After 34 hours, cells were treated with 2.5mM metformin for 8 h. Cells were harvested and total RNA was extracted using the RNeasy Mini Kit (Qiagen). First-strand cDNA synthesis was performed using 1 μg total RNA as a template and SuperScript III First-Strand Synthesis System (Life Technologies) primed with 50 ng of random hexamers. Quantitive real-time PCR was performed using the Bio-Rad SsoFast EvaGreen Supermix (Bio-Rad Laboratories). Gene expression was normalized to the *HPRT* housekeeping gene. The primers for RT-PCR to amplify the genes of interest and HPRT (internal standard) are shown in S12 Table. The sequence (5’->3’) of ATF3 siRNA is as follow: (sense) GGAGGACUCCAGAAGAUGAtt; (antisense) UCAUCUUCUGGAGUCCUCCca.

### Accession numbers

ChIP-seq and RNA-seq data has been made publically available through NCBI (ChIP-seq BioProject ID: PRJNA324846; and RNA-seq BioProject ID: PRJNA324847).

## Supporting Information

S1 Fig Metformin activation of AMPK in Human Hepatocytes. Western blot assay for AMPK α2 (T-AMPK) Thr172 phosphorylation (P-AMPK) in human hepatocytes treated with vehicle control (lanes 1–2), 0.5 mM metformin (lanes 3–4)], 2.5 mM metformin (lanes 5–6) and 10 mM metformin (lanes 7–8) for 4 hours and 8 hours in human hepatocytes.
(PDF)

S2 Fig Comparison of metformin response between our RNA-seq experiment and a previous microarray experiment. **a** Venn diagram showing the overlap of differentially expressed (DE) genes in response to metformin by microarray (light blue) and RNA-seq (purple). **b** Correlation of fold changes between DE genes in either RNA-seq (X-axis) or microarray (Y-axis).
(PDF)

S3 Fig Ingenuity pathway analysis of AMPK-dependent genes shows enrichment for the “AMPK Signaling” canonical pathway. Upregulated and downregulated genes as determined by RNA-seq are colored in red and green, respectively.
(PDF)

S4 Fig Ingenuity pathway analysis of AMPK-independent genes shows enrichment for the “Acute Phase Response Signaling” canonical pathway. Upregulated and downregulated genes as determined by RNA-seq are colored in red and green, respectively.
(PDF)

S5 Fig eQTL analyses. Liver eQTL analyses of rs277070 (A) and rs277072 (B) that are in LD with rs11212617 [R2>0.95 in the Caucasian (CEU) population] show nominally significant associations (P = 0.043 and P = 0.021, respectively) with increased ATM mRNA expression for the treatment response associated SNPs.
(PDF)

S6 Fig Ingenuity pathway analysis of genes found near ATF3-H3K27ac ChIP-seq peaks. Pathway with molecules from the top canonical pathway "EIF2 signaling" added to ATF3, upstream regulators from the IPA analysis of genes nearest the enriched ChIP-seq peaks for ATF3-H3K27ac (*HNF4A*, *MYCN*, *CLOCK*, *TP53* and *MYC*), metformin and gluconeogenesis. Upregulated and downregulated genes as determined by RNA-seq are shown in red and green, respectively.
(PDF)

S7 Fig Enrichment of ATF3 peaks overlapping differentially enriched H3K27ac peaks (denoted DE) compared to a background of all H3K27ac peaks (denoted All). Peaks unique to non-treated (NT) and metformin treated (MET) conditions showed a significant enrichment based on a random permutation test (n = 2000, *P < 0.02), while a significant depletion of ATF3 peaks in common (abbreviated Com) between these conditions was found in DE H3K27ac peaks compared to all peaks.
(PDF)

S1 Table RNA-seq differential expression testing and gene clustering results. (XLSX)

S2 Table Ingenuity pathway analysis for the AMPK-dependent and AMPK-independent genes. Upstream regulator analysis and their target molecules, the DE genes found by RNA-seq. Mechanistic networks for upstream regulators include many DE genes, which further implicate additional molecular pathways related to metformin response. Canonical pathway analysis of AMPK-dependent and AMPK-independent genes show enrichment for “AMPK Signaling” and “Acute Phase Response Signaling”, respectively. Exp Log Ratio showed the log2FoldChange for the genes found by RNA-seq.
(XLSX)

S3 Table Functional annotation enrichment in DE gene clusters by DAVID. (XLSX)

S4 Table ChIP-seq differential enrichment testing for H3K27ac and H3K27me3, and peak clustering for H3K27ac. (XLSX)

S5 Table Functional annotation enrichment in differentially enriched H3K27ac ChIP-seq peaks by GREAT. (XLSX)

S6 Table Differentially enriched H3K27ac ChIP-seq peak locations. (XLSX)

S7 Table Locations, primers and ChIP-seq peak overlaps of promoter and enhancer assays. (XLSX)

S8 Table Association between rs227070 and rs227072 and *ATM* expression in human liver. Within four liver eQTL data sets, linear regression was used to model *ATM* expression levels with adjustment for relevant covariates. Results from the four liver datasets were combined by meta-analysis. *ATM* expression level was determined using microarray and only included patients of European ancestry. The data was coded such that a negative beta means that as the number of minor alleles increases there is decrease in *ATM* expression.
(DOCX)

S9 Table Gene Ontology enrichment analysis of ATF3 metformin unique peaks by GREAT and DAVID. (XLSX)

S10 Table Ingenuity pathway analysis of genes found near ATF3-H3K27ac ChIP-seq peaks. Canonical Pathways from the Ingenuity pathway analysis of the genes nearest the enriched ChIP-seq peaks for ATF3-H3K27ac. Molecules from the top canonical pathway "EIF2 signaling" added to ATF3, Upstream Regulators (HNF4A, MYCN, CLOCK, TP53 and MYC), Metformin, and Gluconeogenesis (shown in S6 Fig).
(XLS)

S11 Table sgRNAs used for CRISPRa. (XLSX)

S12 Table Primers used for qPCR. (DOCX)
