# Supplementary material for: Genomic Characterization of Metformin Hepatic Response
Source: PLoS Genet. 2016 Nov 30;12(11):e1006449. doi: 10.1371/journal.pgen.1006449 (PMC5130177; doi:10.1371/journal.pgen.1006449)
Supplement: S1 Fig — Western blot assay for AMPK α2 (T-AMPK) Thr172 phosphorylation (P-AMPK) in human hepatocytes treated with vehicle control (lanes 1–2), 0.5 mM metformin (lanes 3–4)], 2.5 mM metformin (lanes 5–6) and 10 mM metformin (lanes 7–8) for 4 hours and 8 hours in human hepatocytes. (PDF) [file pgen.1006449.s001.pdf]

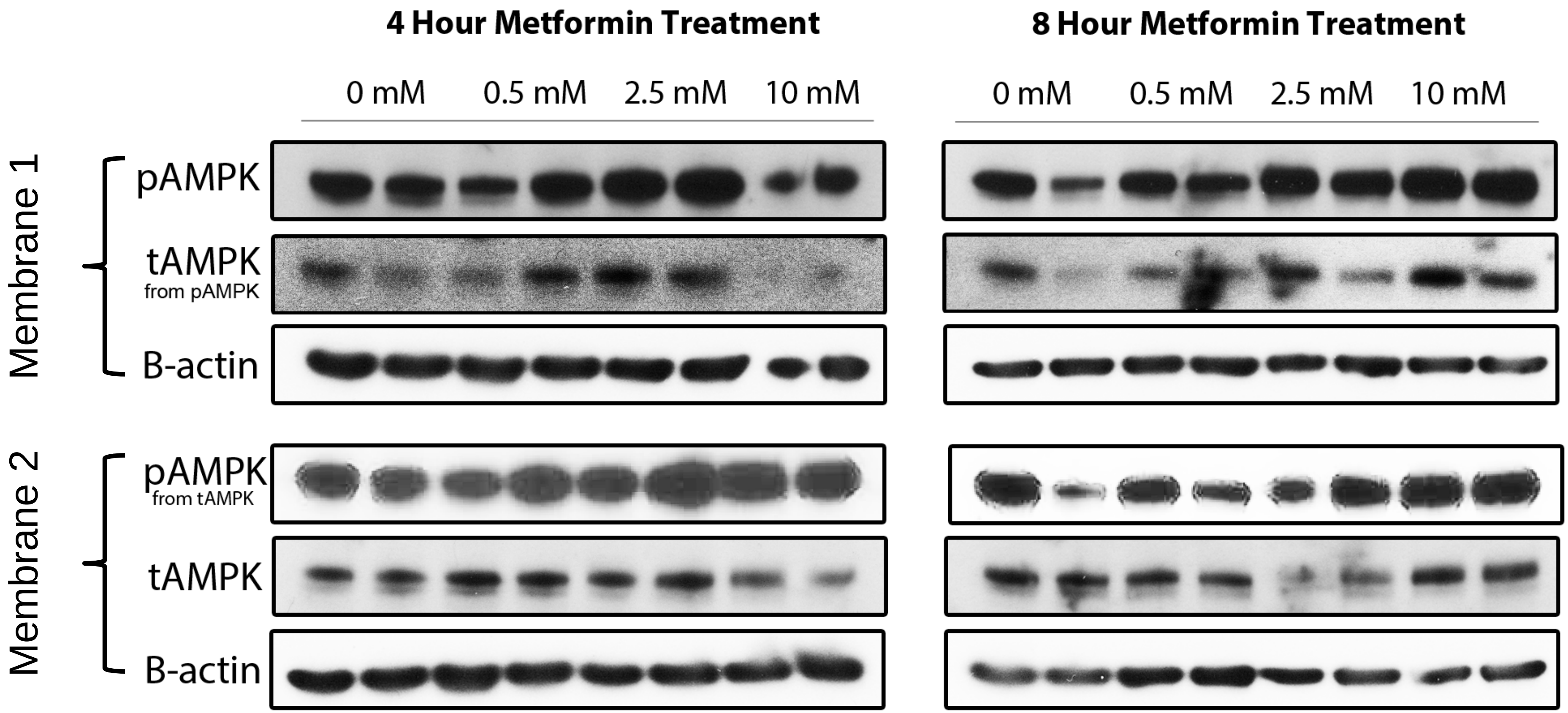
4 Hour Metformin Treatment
8 Hour Metformin Treatment
0 mM
0.5 mM
2.5 mM
10 mM
0 mM
0.5 mM
2.5 mM
10 mM
Membrane 1
pAMPK
tAMPK
from pAMPK
B-actin
Membrane 2
pAMPK
from tAMPK
tAMPK
B-actin
