# Supplementary figures and images for: Genomic Characterization of Metformin Hepatic Response
Source: PLoS Genet. 2016 Nov 30;12(11):e1006449. doi: 10.1371/journal.pgen.1006449 (PMC5130177; doi:10.1371/journal.pgen.1006449)

**A**

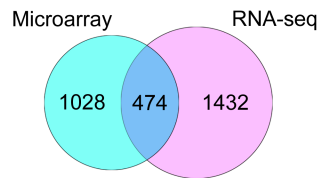

**B**

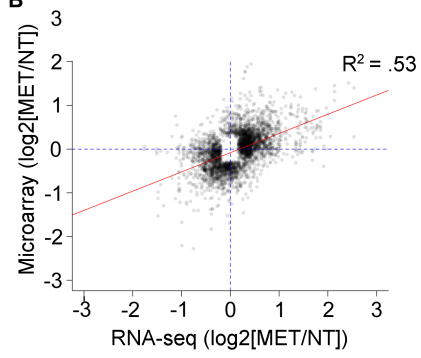

Supplement: S2 Fig — a Venn diagram showing the overlap of differentially expressed (DE) genes in response to metformin by microarray (light blue) and RNA-seq (purple). b Correlation of fold changes between DE genes in either RNA-seq (X-axis) or microarray (Y-axis). (PDF) [file pgen.1006449.s002.pdf]

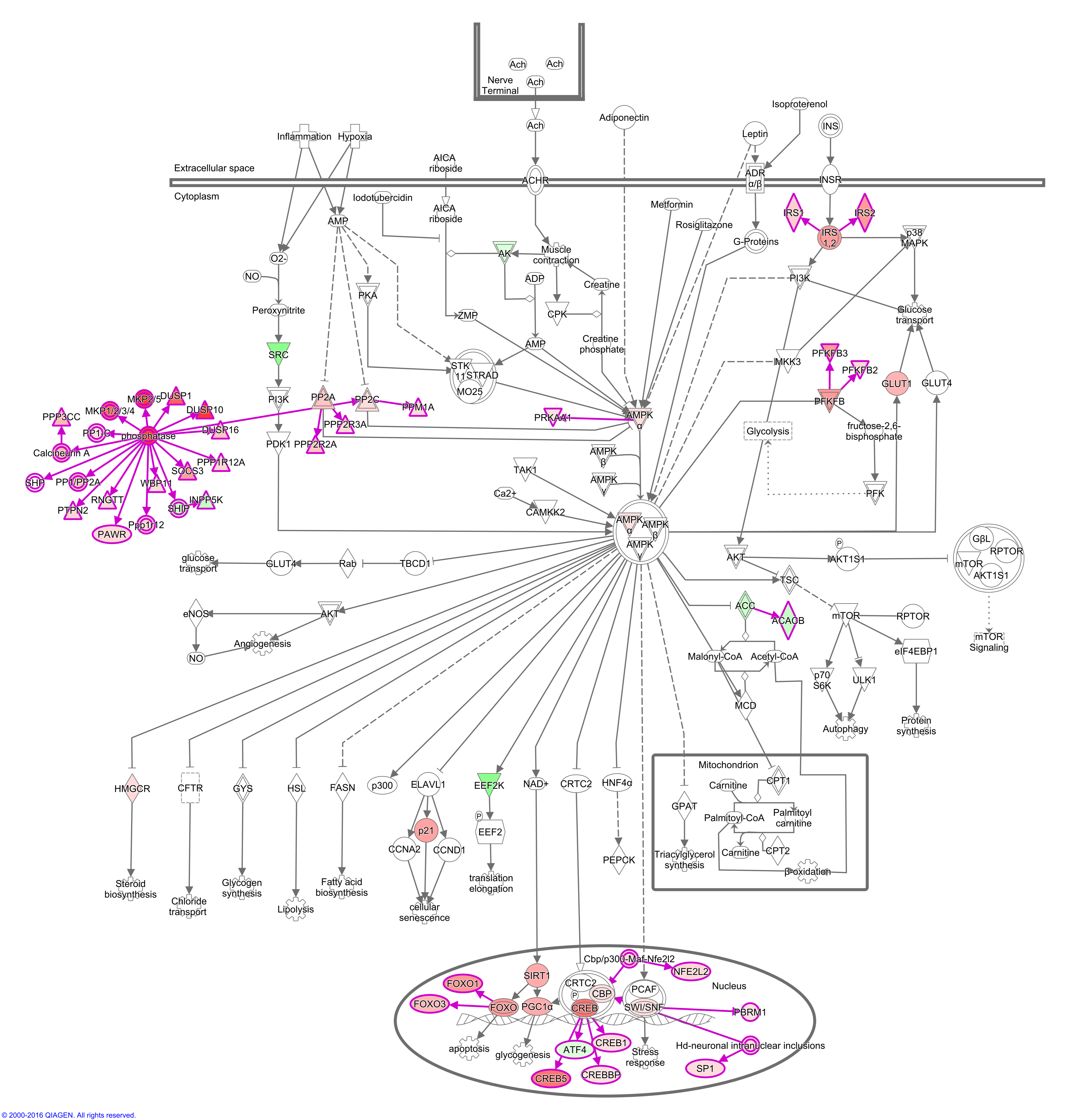

Supplement: S3 Fig — Upregulated and downregulated genes as determined by RNA-seq are colored in red and green, respectively. (PDF) [file pgen.1006449.s003.pdf]

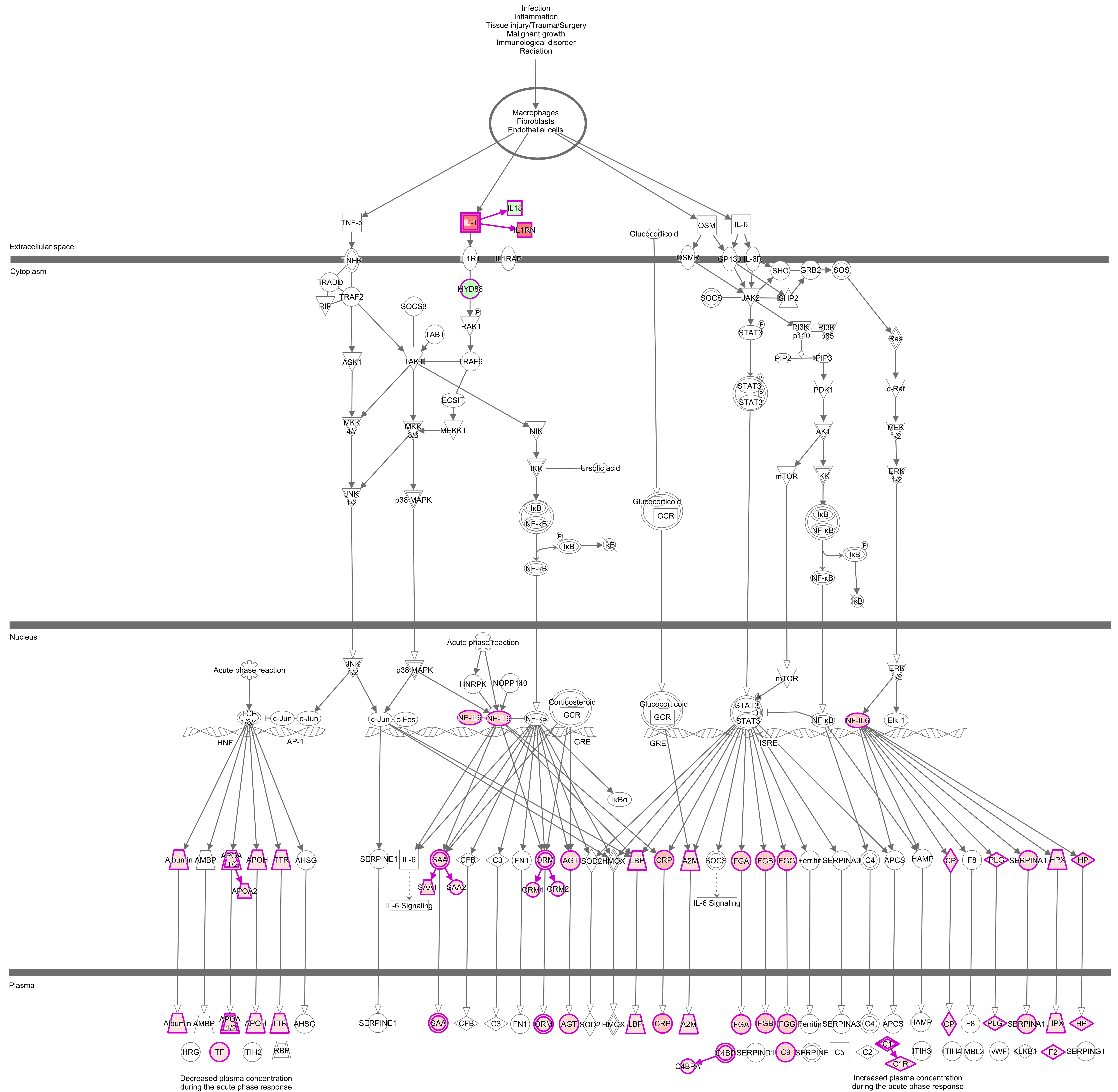

Supplement: S4 Fig — Upregulated and downregulated genes as determined by RNA-seq are colored in red and green, respectively. (PDF) [file pgen.1006449.s004.pdf]

**A**

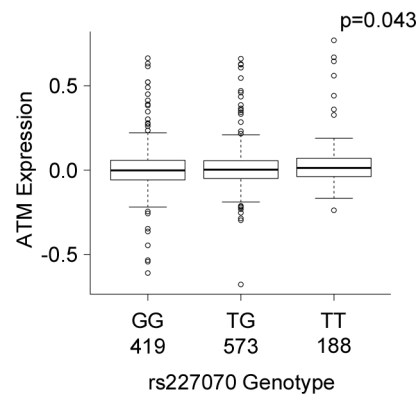

**B**

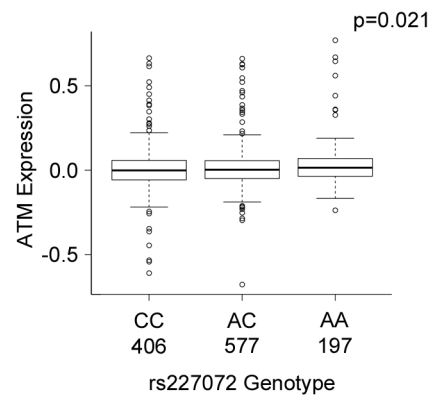

Supplement: S5 Fig — Liver eQTL analyses of rs277070 (A) and rs277072 (B) that are in LD with rs11212617 [R2>0.95 in the Caucasian (CEU) population] show nominally significant associations (P = 0.043 and P = 0.021, respectively) with increased ATM mRNA expression for the treatment response associated SNPs. (PDF) [file pgen.1006449.s005.pdf]

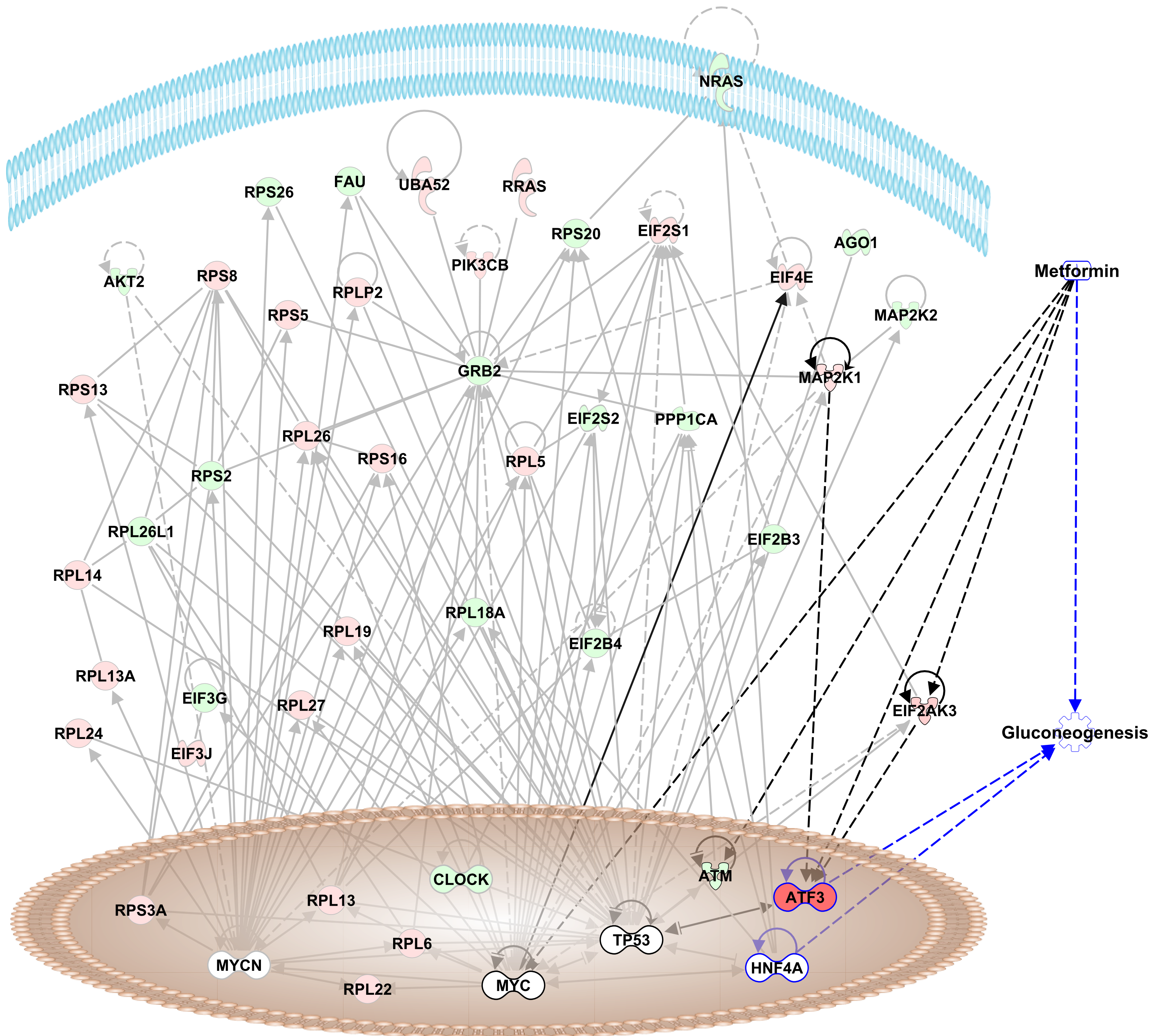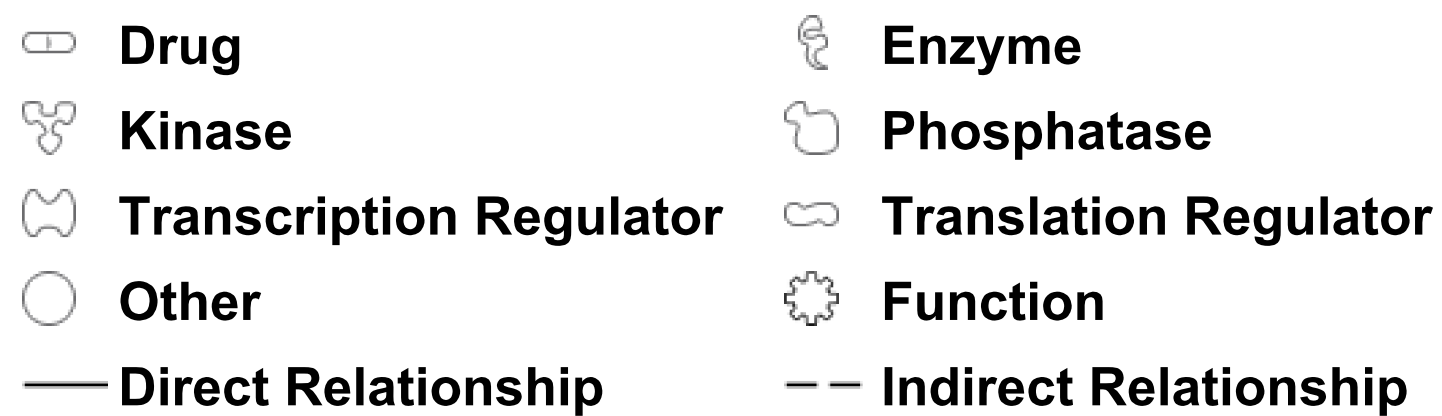

Supplement: S6 Fig — Pathway with molecules from the top canonical pathway "EIF2 signaling" added to ATF3, upstream regulators from the IPA analysis of genes nearest the enriched ChIP-seq peaks for ATF3-H3K27ac (HNF4A, MYCN, CLOCK, TP53 and MYC), metformin and gluconeogenesis. Upregulated and downregulated genes as determined by RNA-seq are shown in red and green, respectively. (PDF) [file pgen.1006449.s006.pdf]

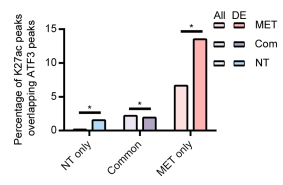

Supplement: S7 Fig — Peaks unique to non-treated (NT) and metformin treated (MET) conditions showed a significant enrichment based on a random permutation test (n = 2000, *P < 0.02), while a significant depletion of ATF3 peaks in common (abbreviated Com) between these conditions was found in DE H3K27ac peaks compared to all peaks. (PDF) [file pgen.1006449.s007.pdf]
