# Supplementary material for: Genomic Characterization of Metformin Hepatic Response
Source: PLoS Genet. 2016 Nov 30;12(11):e1006449. doi: 10.1371/journal.pgen.1006449 (PMC5130177; doi:10.1371/journal.pgen.1006449)
Supplement: S8 Table — Within four liver eQTL data sets, linear regression was used to model ATM expression levels with adjustment for relevant covariates. Results from the four liver datasets were combined by meta-analysis. ATM expression level was determined using microarray and only included patients of European ancestry. The data was coded such that a negative beta means that as the number of minor alleles increases there is decrease in ATM expression. (DOCX) [file pgen.1006449.s015.docx]

**S8 Table.** Association between rs227070 and rs227072 and *ATM* expression in human liver. Within four liver eQTL data sets, linear regression was used to model *ATM* expression levels with adjustment for relevant covariates. Results from the four liver datasets were combined by meta-analysis. *ATM* expression level was determined using microarray and only included patients of European ancestry. The data was coded such that a negative beta means that as the number of minor alleles increases there is decrease in *ATM* expression.

|  | | | | **rs227070** | | **rs227072** | |  |
| --- | --- | --- | --- | --- | --- | --- | --- | --- |
| **Dataset** | **n** | **Expression** | **Genotyping** | **P-value** | **Beta** | **P-value** | **Beta** | **PMID** |
| Set 1 | 149 | Illumina Human Whole Genome-6 v2.0 Expression BeadChip (NCBI GEO accession: GSE39036) | HumanHap300-Duo v2.0 Genotyping BeadChip (NCBI GEO accession: GSE32504) | 0.5237 | -0.0043 | 0.4894 | -0.0046 | 22006096 |
| Set 2 | 164 | Agilent-014850 Whole Human Genome 4x44K gene expression (NCBI GEO accession: GSE25935) | Illumina Human610-Quad v1.0 BeadChip (NCBI GEO accession: GSE26105) | 0.5536 | -0.0027 | 0.4956 | 0.0031 | 21637794 |
| Set 3 | 286 | Agilent Technologies (NCBI GEO accession: GSE9588) | Affymetrix GeneChip Human Mapping 500k genotyping microarray | 0.1075 | 0.0226 | 0.1120 | 0.0222 | 18462017 |
| Set 4 | 581 | Agilent Technologies (NCBI GEO accession: GSE9588) | HumanHap 650Y | 0.2668 | 0.0062 | 0.1488 | 0.0081 | Unpublished |
| Meta | 1180 |  |  | 0.043 |  | 0.0213 |  |  |
