# Supplementary material for: Genomic Characterization of Metformin Hepatic Response
Source: PLoS Genet. 2016 Nov 30;12(11):e1006449. doi: 10.1371/journal.pgen.1006449 (PMC5130177; doi:10.1371/journal.pgen.1006449)
Supplement: S12 Table — (DOCX) [file pgen.1006449.s019.docx]

**S12 Table. Primers used for qPCR.**

| **Target** | **Forward Primer (5’-3’)** | **Reverse Primer (5’-3’)** |
| --- | --- | --- |
| *ATF-3* | CCTCTGCGCTGGAATCAGTC | TTCTTTCTCGTCGCCTCTTTT |
| *PFKFB2* | AGTCCTACGACTTCTTTCGGC | TCTCCTCAGTGAGATACGCCT |
| *PCK1* | TTGAGAAAGCGTTCAATGCCA | CACGTAGGGTGAATCCGTCAG |
| *SIRT1* | TAGCCTTGTCAGATAAGGAAGGA | ACAGCTTCACAGTCAACTTTGT |
| *CRTC2* | GGGGCAGTTGTTTCGACTACC | GGACTGGGGTTCATCACACTT |
| *G6PC* | ACTGGCTCAACCTCGTCTTTA | CGGAAGTGTTGCTGTAGTAGTCA |
| *NR0B2* | CCAATGATAGGGCGAAAGAA | GCTGTCTGGAGTCCTTCTGG |
| *MYC* | CCTACCCTCTCAACGACAGC | CTCTGACCTTTTGCCAGGAG |
| *SERPINE1* | AGTGGACTTTTCAGAGGTGGA | GCCGTTGAAGTAGAGGGCATT |
| *BIRC3* | ATTAATGCTGCCGTGGAAAT | GCCATTCTATTCTTCCGGATT |
| *FBP1* | CTACGCCAGGGACTTTGACC | GGCCCCATAAGGAGCTGAAT |
| *PPP1R15A* | GAAGAGGAAGCTGCTGAGGA | ACAGCCAGGAAATGGACAGT |
| *JUN* | CAGCCCACTGAGAAGTCAAA | CACCAATTCCTGCTTTGAGA |
| *MDM2* | GGCAGGGGAGAGTGATACAGA | GAAGCCAATTCTCACGAAGGG |
| *CEBPB* | GCAACCCACGTGTAACTGTC | AACAAGCCCGTAGGAACATC |
| *CEBPA* | CAAGAAGTCGGTGGACAAGA | GGTCATTGTCACTGGTCAGC |
| *HPRT* | TCCTTGGTCAGGCAGTATAATCC | GTCAAGGGCATATCCTACAACAAA |
